# Soybean Hypocotyls Prevent *Calonectria ilicicola* Invasion by Multi-Layered Defenses

**DOI:** 10.3389/fpls.2021.813578

**Published:** 2022-01-24

**Authors:** Michie Kobayashi, Khin Thuzar Win, Chang-Jie Jiang

**Affiliations:** Institute of Agrobiological Sciences, National Agriculture and Food Research Organization (NARO), Tsukuba, Japan

**Keywords:** soybean, *Glycine max*, *Calonectria ilicicola*, red crown rot, organ-specific infection

## Abstract

In plants, many pathogens infect a specific set of host organs to cause disease, yet the underlying mechanisms remain unclear. Here, we show that inoculation of soybean plants with *Calonectria ilicicola*, the soil-borne causal agent of soybean red crown rot, caused typical disease symptoms of root rot and leaf chlorosis and necrosis. However, the pathogen DNA was only detected in the roots and stem (hypocotyl) base but not other aerial parts of the plants. As we observed vigorous fungal growth in all culture media made of extracts from roots, stems, and leaves, differences in key components including available nutrients did not determine organ-specific infection and reproduction by *C. ilicicola*. Furthermore, inoculation of stems both with and without a surface wound showed that the stems resisted *C. ilicicola* infection *via* both the pre- and post-invasion defense layers. Transcriptomic comparison of roots and stems using RNA-seq analysis further revealed that upon *C. ilicicola* inoculation, a greater expression of genes involved in stress response was induced in the plant stems, including receptor-like kinase, AP2/ERF, MYB, and WRKY. In addition, pathways related to amino acid metabolism were also more upregulated in the stems in response to *C. ilicicola* infection. These results suggest that soybean stems provide *C. ilicicola* resistance, at least in part, by activating an organ-specific defense response.

## Introduction

It is estimated that a plant is exposed to an average of 100 or more potential pathogens in the natural environment (Agrios, 1997). Some of these pathogens can infect all or most organs of their host plants, while others are more limited and cause disease in only one or a few specific organs or tissues, a phenomenon known as organ-specific or structural specific infection (Lacaze and Joly, 2020). For example, *Sclerotinia sclerotiorum* can infect most organs such as roots, leaves, stems, and the reproductive organs of sunflowers (Bolton et al., 2006). The blast fungus *Magnaporthe oryzae*, which generally infects foliar tissues, can also invade the roots of cereals (Dufresne and Osbourn, 2001; Marcel et al., 2010). In contrast, pathogens of powdery mildew disease only infect the epidermis of leaves and green shoots (Lynne, 2016). It remains largely unknown why some pathogens can infect the entire plant while others are restricted to specific organs.

It has been suggested that organ-specific infection is determined by organ-specific resistance in the host plant, such as differences in surface structure, accumulation of secondary metabolites and pathogenesis-related (PR) proteins, and induced resistance, as well as differences in the adaptability of phytopathogens to grow (Balmer and Mauch-Mani, 2013; Lacaze and Joly, 2020). In *Arabidopsis*, *Hyaloperonospora arabidopsidis*, which induces R-gene-mediated defense responses in leaves, can only infect roots (Hermanns et al., 2003). Interestingly, the R gene is expressed in both leaves and roots, indicating that different resistance systems operate downstream of the R gene in roots and leaves (Hermanns et al., 2003). In potato plants, quantitative trait locus (QTL) markers confirmed in foliar resistance to *Phytophthora infestans* are not correlated with tuber resistance (Mayton et al., 2010). In maize, *Ustilago maydis*, the fungal causal agent of smut disease, infects all aerial parts of the plant and locally induces tumor formation (Skibbe et al., 2010). Interestingly, *U. maydis* infection has been shown to induce organ-specific transcriptional changes in both the pathogen and the host (Skibbe et al., 2010). The repertoire of effector genes expressed in different tissues also varies, suggesting that these effectors may be involved in organ-specific infection (Skibbe et al., 2010). Schilling et al. (2014) analyzed 20 presumptive organ-specific *U. maydis* effector genes and showed that nine of them affected virulence only in one of the tested plant organs. Organ-specific infections have also been reported in non-host resistance. For example, *Arabidopsis* is generally a non-host for the rice blast fungus *M. oryzae*; however, two *M. oryzae* isolates (KJ201 and 70–15) were found to be able to infect the roots but not the leaves and stems of *Arabidopsis* with a pathogenic mechanism distinct from that in rice (Park et al., 2009). Interestingly, the fungal hyphae that grew toward the hypocotyl failed to cross the border between the root and hypocotyl tissue (Schreiber et al., 2011).

Red crown rot (RCR) in soybean is caused by the soil-borne pathogenic fungus *Calonectria ilicicola* (anamorph: *Cylindrocladium parasiticum*). *C. ilicicola* was first reported as *Cylindrocladium* black rot on peanuts in the United States in 1965 (Bell and Sobers, 1966), and was found to cause soybean RCR in Japan in 1968 (Krigsvold et al., 1977; Nishi, 1989). RCR causes defoliation and early plant maturity, resulting in yield loss and quality reduction (Roy et al., 1989; Akamatsu et al., 2020). The disease is more severe under clayey or flooding conditions. Yield losses were estimated at 25–30% in Mississippi (Roy et al., 1989), and losses of more than 50% were estimated when the susceptible cultivars were cultivated under humid conditions (Bell et al., 1973; Pataky et al., 1983; Berner et al., 1988; Padgett et al., 2015). Although some differences in RCR resistance have been observed among cultivars (Kim et al., 1998; Win and Jiang, 2021), no true resistance to RCR has been reported.

It has been found that some basal defense responses, such as periderm formation and vessel occlusion, are induced at the site of infection by *C. ilicicola* (Yamamoto et al., 2017), but the detailed infection mechanism remains unclear. *C. ilicicola*-infected soybean plants display typical symptoms of root rot from the early stages of seedling growth, leaf chlorosis (yellowing), and necrosis at the reproductive stages (Roy et al., 1989; Akamatsu et al., 2020). Leaf chlorosis can also be produced by applying culture filtrates of *C. ilicicola* (Kim et al., 2001) and PF1070A, a cyclic peptide consisting of four amino acids identified as a phytotoxin of the *C. ilicicola* fungus (Ochi et al., 2011). However, it is still unclear whether the disease symptoms in leaves are caused by *C. ilicicola* infection or by the transmission of the *C. ilicicola*-produced phytotoxin from roots to the aboveground parts of plants.

This study aimed to elucidate the organ-specificity of *C. ilicicola* infection and the underlying mechanisms in soybean plants. We show that *C. ilicicola* exclusively infects roots, whereas stems prevent infection by activating distinct defense mechanisms. Our findings have important implications for understanding pathogenic mechanisms of red crown rot and the immune system of soybean.

## Materials and Methods

### Plant Material and Growth Conditions

Seeds of the soybean cultivar Enrei were pre-conditioned in a moisture-saturated plastic box for 24–48 h at 25°C. The seeds were then sown in commercially available pre-fertilized and granulated soil (Nippi No. 1, Nippon Hiryo, Tokyo, Japan) in 65-mm^2^ plastic pots with a depth of 50 mm (180 ml) and a drainage hole. Five seeds were sown per pot, and the top of the pot was covered with a 2-mm layer of pre-fertilized peaty soil Supermix-A (Sakata Seed Corporation, Yokohama, Japan). Plants were then grown in a climate-controlled plant growth chamber (Nippon Light Metal Company, Ltd., Tokyo, Japan) under 16-h light conditions at 25°C and 50% relative humidity (RH). All the soils used in this study were autoclaved one day before seed-sowing to eliminate any effects from other soil pathogens.

### Pathogen Culture and Inoculation

*Calonectria ilicicola* (isolate UH2-1) was kindly provided by Dr. Sunao Ochi (Research Center for Agricultural Information Technology, NARO, Japan), which was isolated from RCR-diseased soybean roots from Sasayama, Hyogo (Jiang et al., 2020). Fungal mycelia were grown on potato dextrose agar (PDA) plates at 25°C for 1–2 weeks or until fungal mycelial growth reached the edges of the Petri plates.

**FIGURE 1:**
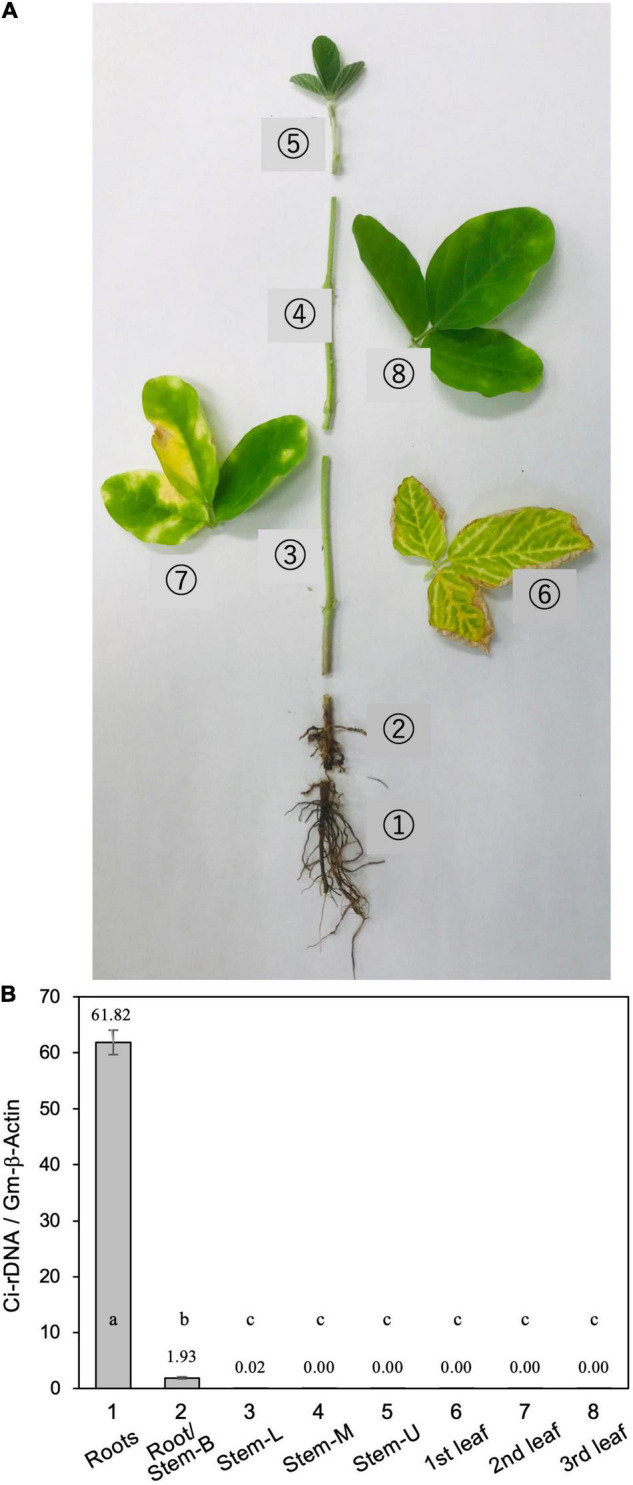
**(A)** Typical disease symptoms of red crown rot in a diseased soybean plant. The photograph was taken 5 weeks after inoculation with *Calonectria ilicicola* (isolate UH2-1). Eight different parts (1–8) of from three diseased plants were sampled for measurement of relative fungal growth: (1) roots, (2) roots and stem base (Roots/stem-B), (3–5) lower (Stem-L), middle (Stem-M), and upper (Stem-U) portions of stem, and (6–8) first, second, and third trifoliate leaves. **(B)** Relative fungal growth in different parts of diseased soybean plants. Values are means ± SD, *n* = 3 plants. Different letters indicate significant differences (Turkey’s multiple comparison test, *p* < 0.01).

For soil inoculation, five to eight pieces (∼5-mm cubes) of PDA with vigorously growing *C. ilicicola* mycelia were placed in a 500-ml flask containing 200 g of wheat bran-vermiculite medium (wheat bran/vermiculite/water 1:1:3, w/w/v) and incubated at 26°C for 10–14 days until the fungal mycelia fully covered the medium (Ochi and Nakagawa, 2010). This culture was used as inoculum, and an inoculum-soil mixture was prepared by mixing the inoculum with Nippi No. 1 soil to generate a concentration of 1% (w/v). The soil mixture was then filled into large plastic pots (12 × 12 × 20 cm, 1,500 ml) into which five seeds were sown per pot as described above (Jiang et al., 2020). After 5 weeks of inoculation, three diseased plants were removed from the plant pots, and the following parts of each plant were sampled and measurement for relative fungal growth (Figure 1A): roots, stem base, stem (lower, middle, and upper portions), and leaves (first, second, and third trifoliate leaves).

**FIGURE 2:**
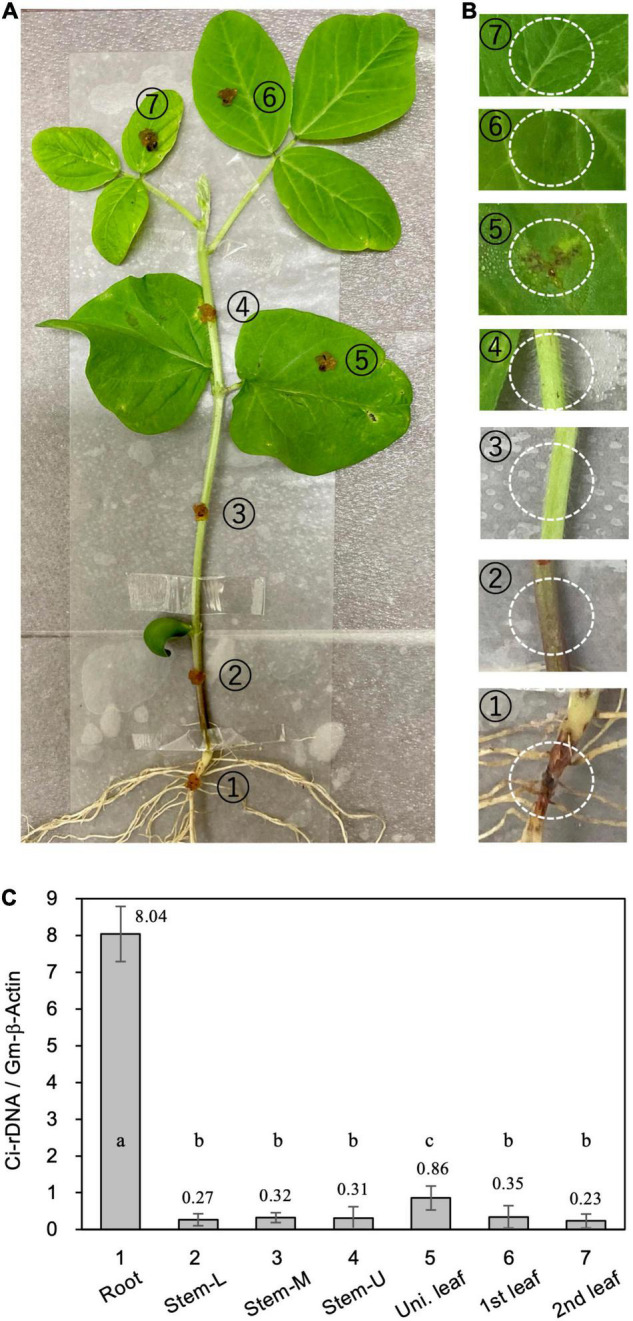
Relative fungal growth in different parts of soybean plants. **(A)** *Calonectria ilicicola* was inoculated to a 5-mm-diameter spot on (1) radical root, (2–4) lower (Stem-L), middle (Stem-M), and upper (Stem-U) portions of stem, and (5–7) unifoliate (Uni. leaf), first and second trifoliate leaves (1st and 2nd leaf) of 4-week-old soybean plants. **(B)** Images of inoculated spots (white dotted circles) 6 days after inoculation. **(C)** Relative fungal growth (Ci-rDNA/Gm-b-Act1) in the inoculated parts of soybean plants. Values are means ± SD, *n* = 5 plants. Different letters indicate significant differences (Turkey’s multiple comparison test, *p* < 0.05).

For direct inoculation of the roots and stems, 4-week-old soybean seedlings were removed from the pots and the roots were washed gently with running tap water to remove adhering soil. The seedlings were then placed in a moisture-saturated plastic box. *C. ilicicola* mycelia from the PDA plates were homogenized by passage through a syringe three times (agar inoculum), and then applied to a 5-mm-diameter spot on seven different plant parts: radical root, stem (lower, middle, and upper portions) and leaves (unifoliate, first and second trifoliate leaves) (Figure 2A).

For the inoculation of wounded organs, the surfaces of hypocotyls and radicle roots of 4-day-old soybean seedlings were cut longitudinally (5-mm length, 0.5-mm depth) with a needle, and the agar inoculum was then applied to the cut sites (Figure 4A). The inoculated seedlings (in the plastic box) were placed in a plant-growth chamber under 16-h light conditions for several days as indicated in the corresponding figures.

**FIGURE 3:**
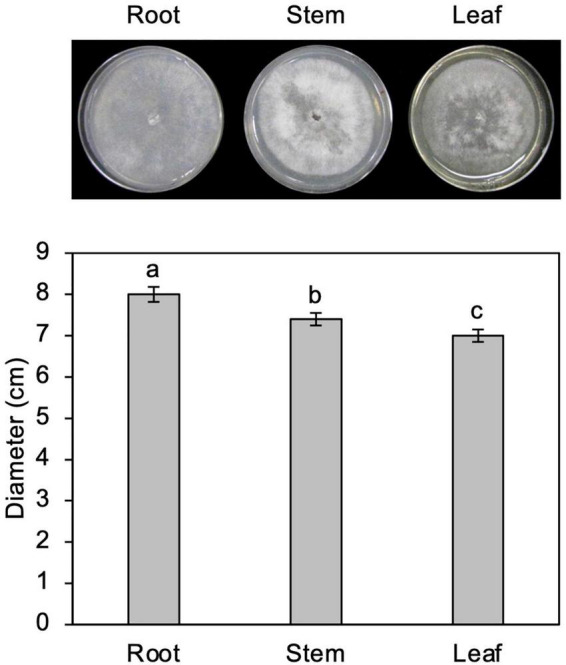
Growth of *Calonectria ilicicola* in culture media containing extracts from roots, stems, and leaves. Representative images **(upper panel)** and average mycelial growth **(lower panel)** were taken after 2 weeks of culturing. Different letters indicate significant differences (Turkey’s multiple comparison test, *p* < 0.05).

**FIGURE 4:**
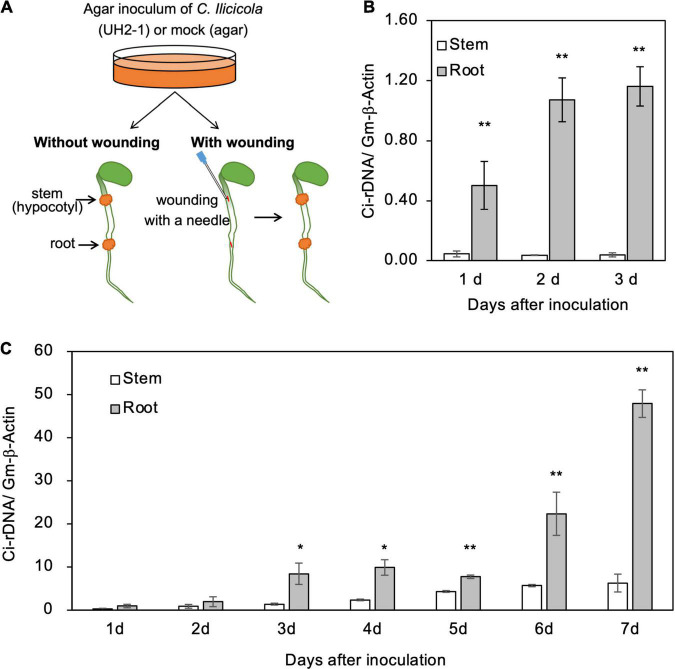
Relative fungal growth in roots and stems of soybean seedlings. **(A)** Illustration of method for direct inoculation of *Calonectria ilicicola* to the roots and stems. Agar inoculum was applied to a 5-mm diameter spot on the radical roots and stems (hypocotyl) of 4-day-old seedlings with or without wounding. **(B,C)** Relative fungal growth (Ci-rDNA/Gm-β-Actin) in non-wounded **(B)** and wounded **(C)** roots and stems. Asterisks indicate significant differences (non-parametric *t*-test, *, *p* < 0.05; **, *p* < 0.01).

The agar inoculum was removed from the plant tissue surfaces by brushing under running tap water, and 10-mm long/diameter sections of the plants, including the inoculated spots, were sampled for measuring relative fungal growth. Homogenized PDA without mycelium was used for mock inoculation, and three replicates of four seedlings were used for each inoculation.

### Preparation of Soybean Extract Culture Media

Three-week-old soybean seedlings were removed from the plant pots, and their roots, stems, and leaves were cut off. The roots were washed gently with running tap water to remove adhering soil. Two grams of each sample were frozen and ground to powder in liquid nitrogen with a pestle and mortar, and suspended in 100 ml of distilled water (2% extract) in a 300-ml flask. After adding Bacto-Agar (1.5 g) (Difco Laboratories, Detroit, MI, United States), the flask was autoclaved and the soybean extract culture media poured into sterile Petri dishes and allowed to set at room temperature. Five plates of each medium were inoculated with a small mycelial plug on the plate center, and then incubated at 25°C for 2 weeks. The fungal growth rate was represented as the colony diameter (cm).

### Measurement of *Calonectria ilicicola* Accumulation by Quantitative Real-Time Polymerase Chain Reaction

Relative fungal growth was measured by quantitative real-time polymerase chain reaction (qPCR). All measurements were performed with three biological replicates, and each replicate consisted of five plants. Genomic DNA was extracted from plant tissues using MagExtractor (Toyobo, Osaka, Japan) following the manufacturer’s instructions. Real-time PCR was run on a Thermal Cycler Dice TP800 system (Takara Bio Inc., Otsu, Japan) using SYBR premix Ex Taq mixture (Takara) with cycles of 95°C for 5 s, 55°C for 20 s, and 72°C for 20 s. Three technical replicates were used for each biological replicate sample. The PCR primers used were as follows: (1) primers targeting the intergenic spacer region of the *C. ilicicola* rDNA, CiIGSF (forward) = 5′-TCCATTGCCTCTATTTATCCTGC-3′, and CiIGSR (reverse) = 5′-GCGTAAAGATTTTCCAACCCG-3′ (Ochi and Kuroda, 2021); (2) primers for soybean β-Actin gene (Gm-β-Actin; Glyma.15G050200), Gm-β-ActinF (forward) = 5′-GAGCTATGAATTGCCTGATGG-3′, and Gm-β-ActinR (reverse) = 5′-CGTTTCATGAATTCCAGTAGC-3′ (Sugano et al., 2013). Relative fungal growth was expressed as *C. ilicicola* rDNA amplification folds relative to the host actin gene amplification (Jiang et al., 2020).

### RNA-Seq Analysis

Total RNA from mock and *C. ilicicola*-infected radicle roots and stems (non-wounded) was extracted using the RNeasy Plant Mini Kit (Qiagen) following the manufacturer’s instructions. The Illumina NovaSeq platform was used for RNA-Seq. cDNA library construction and sequencing were performed by Takara Bio Inc. (Japan).

Differential expression analysis was performed using the HISAT2-featureCounts-edgeR pipeline. HISAT2 (Kim et al., 2015) was used to align the generated reads to the Phytozome13 *Glycine max* Wm82.a2.v1 reference genome (Schmutz et al., 2010). We then used featureCounts (Liao et al., 2014) for quantification and edgeR (Robinson et al., 2009) for differential analysis (FDR < 0.05). The consistency among replicate samples was analyzed by multidimensional scaling (MDS) plot using edgeR (Supplementary Figure 1). Gene ontology (GO) enrichment analysis was performed using agriGO v2.0 (Tian et al., 2017^1^) with the Plant GO Slim option (FDR < 0.05). Kyoto Encyclopedia of Genes and Genomes (KEGG) pathway enrichment analysis was performed using DAVID functional annotation tool (Huang et al., 2009^2^).

The raw data of RNA-Seq has been deposited to NCBI BioProject database with the accession no PRJNA781415.

### Gene Expression Analysis

A 500 ng of total RNA was reverse transcribed to cDNA using ReverTra Ace (Toyobo, Osaka, Japan) according to the manufacturer’s instructions. Quatitative reverse trascription-PCR (qRT-PCR) was performed using soybean β-Actin gene (Gm-β-Actin; Glyma.15G050200) as an internal reference for normalization (Sugano et al., 2013). The primer sequences used for qRT-PCR are listed in Supplementary Table 1.

### Experimental Design and Statistical Analysis

All experiments were conducted with three replicates, each consisting of five plants per pot. Analysis of variance (ANOVA) was performed for all measurements using the Statistical Tool for Agriculture Research (STAR) Version 2.0.1 (International Rice Research Institute, Philippines).

## Results

### *Calonectria ilicicola* Was Detected Exclusively in the Roots

*Calonectria ilicicola* inoculation at seed sowing resulted in severe root rot during the early growth stages of soybean plants, followed by the appearance of brown necrosis on the lower leaves and yellow chlorosis in the upper leaves (Figure 1). Real-time PCR analysis of ribosomal DNA (rDNA) of *C. ilicicola* in eight different parts of the diseased soybean plants detected the pathogen DNA only in the roots and stem base but not in the stem (lower, middle, and upper portions) or leaves (first, second, and third trifoliate leaves) (Figure 1).

We questioned whether the absence of *C. ilicicola* in non-root organs was simply due to their physical isolation from the inoculum or, alternatively, the non-root organs could actively protect themselves from infection. To clarify this, we inoculated seven different parts of soybean plants by directly applying the agar inoculum of *C. ilicicola* culture: (1) radical root, (2–4) lower (Stem-L), middle (Stem-M) and upper (Stem-U) portions of stem, and (5–7) unifoliate (Uni. leaf), first and second trifoliate leaves (1st and 2nd leaf) of 4-week-old soybean plants (Figure 2A). Six days after inoculation (dpi), a reddish infectious lesion on the roots and some small black lesions on unifoliate leaves were observed (Figure 2B). The qPCR measurement detected *C. ilicicola* mainly in the roots and a small amount in the unifoliate leaves (Figure 2C). No appreciable *C. ilicicola* growth was detected in stem and trifoliate leaves (Figure 2C).

### *Calonectria ilicicola* Showed a Vigorous Growth in Culture Media Containing Different Organ Extracts

To test the possibility that organs other than roots are nutritionally unfavorable for fungal growth, *C. ilicicola* was grown in culture media containing extracts from roots, stems, and leaves. As shown in Figure 3, the fungus showed vigorous growth in all the extract media, although approximately 6 and 10% reductions in growth were observed in stem and leaf extracts, respectively, compared to the root extract. The *C. ilicicola* mycelia fully covered the extract media in 2 weeks, which was comparable to that in PDA.

### Stems Resisted *Calonectria ilicicola* Infection at Both Pre- and Post-invasion Defense Layers

To get some insights into the mechanisms of resistance to *C. ilicicola* in stems (Figures 1, 2), we applied the agar inoculum to the radicle roots and stems (hypocotyls) of 4-day-old soybean seedlings (Figure 4A). As a result, a significant fungal growth was observed in the roots from 1 dpi and increased at 2–3 dpi (Figure 4B). In contrast, in the stems, fungal growth was detected only in trace amounts at 1–3 dpi (Figure 4B). These results indicate that the stems were capable of repelling *C. ilicicola* infection at pre-invasion levels.

Plants have evolved multiple layers of defense to protect themselves from various pathogen attacks, and the physical and chemical barriers on their surfaces are considered the first layer of defense. To address whether soybean stems can self-protect beyond this surface defense layer, the surfaces of the roots and stems of the seedlings were experimentally wounded and then inoculated with the agar paste of the *C. ilicicola* culture (Figure 4A). *C. ilicicola* growth was subsequently observed in both the roots and stems at 1 dpi and then increased until the end of the experiment at 7 dpi (Figure 4C). More fungal growth was detected at 1–3 dpi in the wounded seedlings than under the non-wounded inoculation conditions (Figure 4B). There was no significant difference in fungal growth between roots and stems at 2 dpi; however, it became evident that the fungal growth in roots was significantly greater than that in stems from 3 dpi onward (Figure 4C). These results suggest that soybean stems can also combat *C. ilicicola* infection at the post-invasion level.

### Stems Showed a Stronger Immune Response to *Calonectria ilicicola* Infection

Total RNA-seq analysis was performed to analyze the transcriptome in the roots and stems. Multidimensional scaling (MDS) plot of RNA-seq data showed a clear separation into four subgroups (the roots and stem with or without *C. ilicicola* inoculation) (Supplementary Figure 1), indicating significant differences in the global gene expression profiles between the roots and stems in response to *C. ilicicola* infection. Furthermore, the transcriptome data from RNA-seq was validated by qRT-PCR expression analysis of 10 selected DEGs (Supplementary Table 1). Based on the log2 fold-change of these genes, the RNA-seq data showed a significant linear correlation with the qRT-PCR results (Pearson coefficient *R*^2^ = 9667, *P* < 0.01) (Supplementary Figures 2, 3), indicating a good accordance between the two measurements.

**FIGURE 5:**
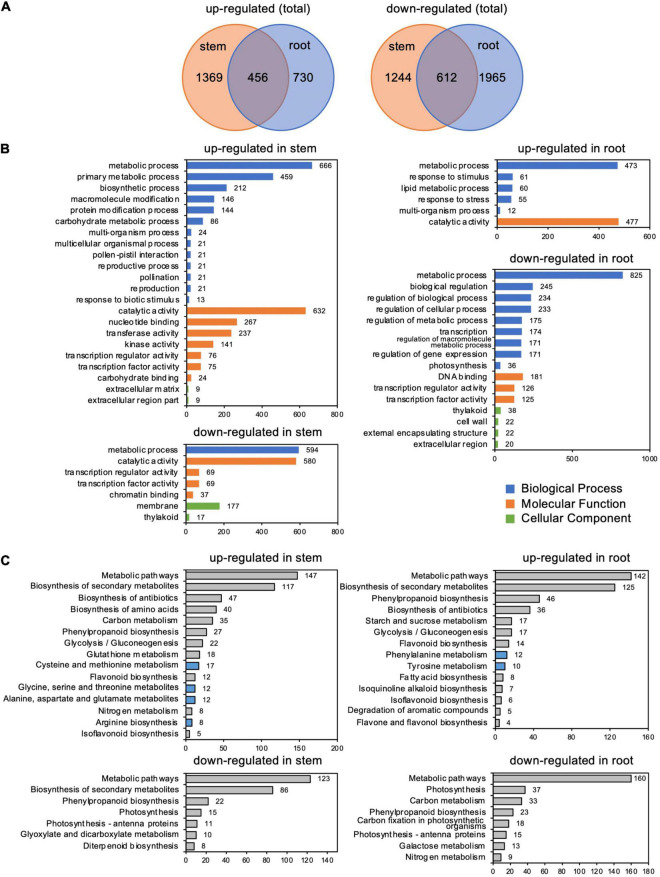
Differentially expressed genes (DEGs) in stems and roots inoculated with *Calonectria ilicicola* at 24 h post-inoculation (hpi). **(A)** Venn diagram showing the number of significant DEGs (FDR < 0.05, >twofold) and shared genes in stems and roots. **(B)** GO term enrichment analysis of identified DEGs. The horizontal axis shows the number of genes classified into each GO term. **(C)** KEGG pathway enrichment analysis of identified DEGs.

RNA-seq analysis identified 3,681 and 3,763 differentially expressed genes (DEGs) in stems and roots, respectively, whose expression changed more than twofold in response to *C. ilicicola* infection. A comparison of these DEGs between stems and roots showed that 1,825 and 1,186 DEGs were upregulated, and 1,856 and 2,577 DEGs were downregulated, in stems and roots, respectively (Figure 5A and Supplementary Table 2). In addition, 456 and 612 DEGs were upregulated and downregulated in the stems and roots, respectively (Figure 5A). GO enrichment analysis by Plant GO Slim showed that 22 terms were enriched in the genes upregulated in the stems, which contrasted with six terms in the roots. Among the downregulated genes, seven terms were enriched in the stems and 16 terms were enriched in the roots (Figure 5B).

**FIGURE 6:**
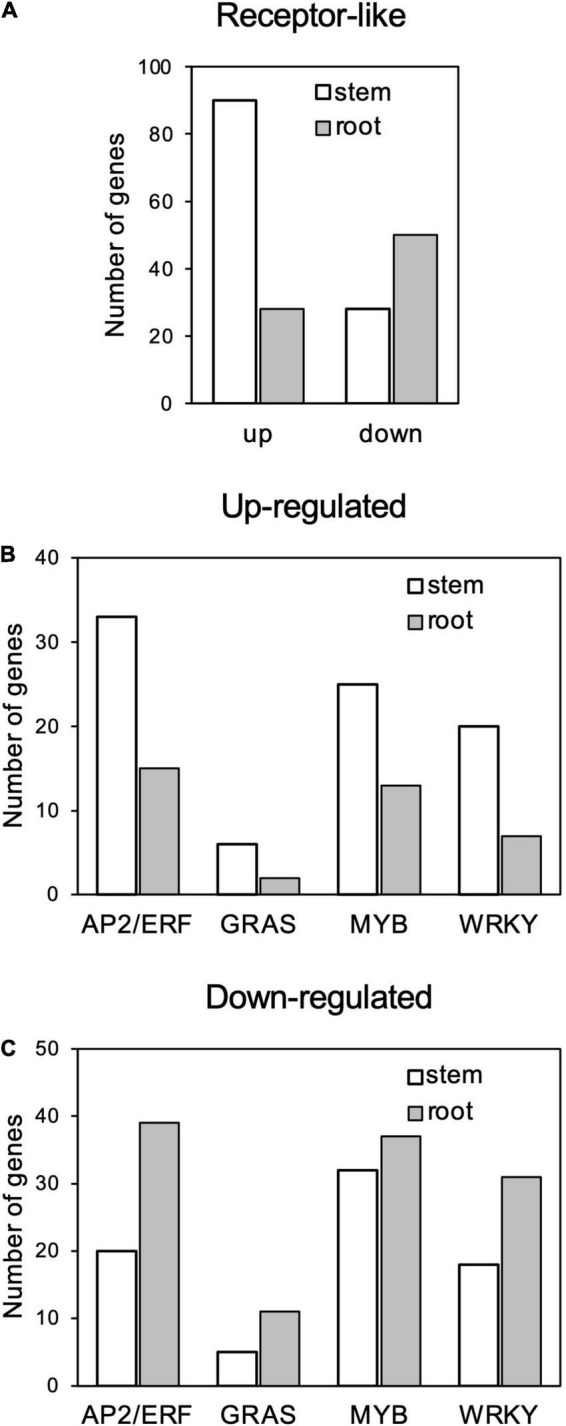
Number genes of receptor-like proteins **(A)** and AP2/ERF, GRAS, MYB, and WRKY transcription factors **(B)** up- and **(C)** downregulated in response to *Calonectria ilicicola* infection in stems and roots.

Among the GO terms enriched in upregulated genes in the stems, many genes overlapped in the categories of “multi-organism process,” “reproduction,” “pollination,” “reproductive process,” “pollen-pistil interaction,” and “multicellular organismal process”; and 21 genes for receptor-like kinases annotated to receptor kinase or S-locus protein kinase were common to all of these categories (Supplementary Tables 3, 4). In addition, many genes overlapped in the categories “protein modification process,” “macromolecule modification,” and “kinase activity”; and 133 genes were common to all of these categories. Many of these genes encode proteins that are annotated to receptors, such as receptor-like kinases and LRR kinases (Supplementary Tables 3, 5). Notably, the number of upregulated genes annotated as receptor-like proteins (Supplementary Table 2) was higher in stems than in roots (Figure 6A).

Gene ontology terms related to transcription regulation, such as “transcription regulator activity” and “transcription factor activity,” were enriched for both up- and downregulated genes in the stems in contrast to only downregulated genes in the roots (Figures 6B,C). Of particular interest was that more genes involved in stress response, such as AP2/ERF, GRAS, MYB, and WRKY, were upregulated in the stems (Figure 6B and Supplementary Table 2) while downregulated in the roots (Figure 6C and Supplementary Table 2).

Kyoto Encyclopedia of Genes and Genomes pathway analysis indicated that the most abundant DEGs were enriched in “Metabolic pathway” and “Biosynthesis secondary metabolites” in both the roots and stems (Figure 5C and Supplementary Table 6). Of particular interest, the upregulated DEGs were also enriched in pathways related to amino acid metabolism: four items in stems, including “cysteine and methionine metabolism,” “alanine, aspartate, and glutamate,” “glycine, serine, and threonine metabolism,” and “arginine biosynthesis” DESs; and two items in the roots, including “phenylalanine metabolism” and “tyrosine metabolism” (Figure 5C and Supplementary Table 6).

## Discussion

We showed that *C. ilicicola* mainly colonized the roots and not the other aerial parts of soybean plants (Figure 1). This observation demonstrates a strong natural tendency of this pathogen to infect specific organs. The root-colonized fungal hyphae likely invaded the hypocotyl tissue to some extent, but it was strictly restricted within the stem base (Figure 1). Direct inoculation of *C. ilicicola* to different parts of soybean plants confirmed that *C. ilicicola* mainly infects the roots (Figure 2). A small amount of *C. ilicicola* was also detected in the old unifoliate leaves, which may be associated with programmed cell death during leaf aging and senescence. Cell death can cause tissue damage and nutrient leakage and thus benefit pathogen growth (Zhang et al., 2020). Taken together, these results suggest that the stem (hypocotyl) and other aboveground organs are equipped with strong defense mechanisms against *C. ilicicola*. Furthermore, our results revealed that the stems provide both pre- and post-invasion defense layers (Figure 4) involving defense mechanisms that are distinct from the roots (Figures 5, 6).

The infection of soybean seedlings by *C. ilicicola* caused typical symptoms of chlorosis and necrosis in leaves in addition to root rot (Figure 1). However, no pathogen DNA was detected in diseased leaves. This result indicates that leaf symptoms are caused by factors other than the direct infection and proliferation of *C. ilicicola*. One possible explanation may come from the previous studies of Kim et al. (2001) and Ochi et al. (2011), who showed that the disease symptoms in leaves can be reproduced by the application of phytotoxic compounds from the culture filtrate of *C. ilicicola*. These results, together with ours, suggest that the symptoms observed in aboveground organs may be ascribed to the distal transmission of *C. ilicicola* phytotoxins from the infected roots, causing chlorotic and necrotic cell death. In contrast, the fungal pathogen *Fusarium oxysporum*, the root-infecting causal agent of wilt disease in several plant species, first colonizes the roots and then spreads to the leaves, where it causes disease symptoms (Lyons et al., 2015).

It has been reported that there are significant differences in nutrients and antimicrobial compounds between different plant organs, which may correlate with organ-specific infections. Analysis of soluble aromatic compounds in extracts from *Arabidopsis* roots and leaves revealed that flavanol glycosides and sinapoyl esters—the most abundant soluble phenylpropanoids in leaves—were absent in roots, whereas roots contained three prominent phenylpropanoids (coniferin, syringing, and scopolin) not found in leaves (Bednarek et al., 2005). Constitutive accumulation of antimicrobial compounds is a type of preformed defense system in plants, and their distribution, is often tissue-specific (Osbourn, 1996). In our study, we observed some differences in fungal growth among the extracts, with the highest growth rate in the root extract, which may account for the differential infection between the roots and other organs. However, this minor difference is unlikely to explain the exclusive organ-specific infection and proliferation in the roots.

In contrast to vigorous fungal growth in the roots, direct inoculation of *C. ilicicola* to the radicle root and stem resulted in almost no fungal growth in stems (Figure 4B). On the other hand, the wounded stems allowed fungal growth to some extent but were suppressed to a significantly lower level compared to the radicle roots (Figure 4C). This suggests that stems protect themselves from fungal invasion at both the pre- and post-invasion levels. It is plausible that the cuticular layers of stems serve as the first physical barrier to pre-invasion by *C. ilicicola*. The cuticular layer is formed only in the aboveground parts of plants and at the root tips (Berhin et al., 2019). It has been reported that the fungal pathogen *Nectria haematococca* (anamorph: *Fusarium solani* f. sp. *pisi*) typically infects the roots of peas and not the leaves, but can infect leaves when plants are wounded (Schäfer and Yoder, 1994). Similarly, in the interaction between *Phytophthora palmivora* and barley, leaves can be infected when they are wounded (le Fevre et al., 2016). Our observation of *C. ilicicola* resistance in the wounded stems of soybean suggests a strong defense response even after penetration of the fungus into the stem tissues.

It has been reported that different organs have different responses to defense signals. Badri et al. (2008) reported that treatment of *Arabidopsis* roots with defense-related plant hormones, such as salicylic acid and methyl jasmonate, induced a different set of genes compared to the leaves. In rice roots inoculated with *M. oryzae*, defense-related genes involved in leaves were transiently induced by infection, but their expression was subsequently suppressed (Marcel et al., 2010). In our study, the soybean stems exhibited a transcription profile apparently distinct from the roots in response to *C. ilicicola* infection, which may make a crucial contribution to the stem defense (Figure 5). Notably, the expression of genes involved in stress response, such as receptor-like genes and transcription factors including AP2/ERF, GRAS, MYB, and WRKY, was highly increased in the stems compared to the roots (Figure 6). AP2/ERF is a transcription factor involved in ethylene signaling. By activating defense responses, it has been reported that pre-treatment of soybean with ethephon, which is metabolized into ethylene in plants, enhances resistance to root and stem rot caused by *Phytophthora sojae* (Sugano et al., 2013) as well as sudden death syndrome caused by soil-borne *Fusarium virguliforme* (Abdelsamad et al., 2019). The GRAS family encodes plant-specific transcription factors, and CIGR1 and GIGR2, the GRAS genes from rice, are rapidly induced by the *N*-acetylchitooligosaccharide elicitor (Day et al., 2003). Overexpression of the poplar GRAS gene, PeSCL7, which is induced by high salt and drought stress, results in drought and salt tolerance in *Arabidopsis* (Ma et al., 2010). Some MYB transcription factors have also been shown to positively (Yi et al., 2010; Chu et al., 2017) or negatively (Liu et al., 2013; Yan et al., 2015) regulate isoflavonoid biosynthesis in soybean. WRKY is a superfamily of transcription factors that play multiple roles in plant physiological processes, including biotic (Eulgem et al., 2000) and abiotic (Zhou et al., 2008; Dong et al., 2019) stress responses in soybean. Induction of defense responses is triggered by the sensing of pathogen invasion by receptors such as pattern-recognition receptors (PRRs) and nucleotide-binding domain leucine-rich repeat proteins (NLRs). Organ-specific expression has been reported in both PRRs (Bartels et al., 2013; Ahmadi et al., 2016) and NLRs (Park et al., 2005; Gao and Bradeen, 2016). In the case of the soybean–*C. ilicicola* interaction, it is tempting to speculate that fungal invasion is perceived by these receptors in the stem, which in turn activates defense responses that include ethylene signaling pathways.

The KEGG pathway analysis revealed an enrichment of upregulated DEGs in pathways related to amino acid metabolism: four items for nine amino acids (cysteine, methionine, alanine, aspartate, glutamate, glycine, serine, threonine, and arginine) in stems, and two items for two amino acids (phenylalanine and tyrosine) in the roots, respectively (Figure 5C). These results suggest that amino acid metabolism may play important roles in response to *C. ilicicola* in soybean plants. Amino acids are in fact precursors of many defense-related phytoalexins, so their metabolism has a profound impact on the plant immune system (Zeier, 2013; Qiu et al., 2020; Sun et al., 2020). In support, recent studies have shown that exogenous applications of glutamate (Kadotani et al., 2016; Yang et al., 2020), histidine (Seo et al., 2016; Yariyama et al., 2019), and lysine (Liu et al., 2021) enhanced disease resistance in various plant species. In soybean, it has been reported that foliar application of cysteine, glutamate, and threonine significantly reduced the disease severity of bacterial pustule caused by *Xanthomonas citri* pv. *glycines* (Pluemjit et al., 2020).

## Conclusion

We have reported the organ-specific infection and reproduction of *C. ilicicola* on soybean roots. The fungus grew well on media made from roots, stems, or leaves, indicating that differences in key components among organs, such as nutrients, are not the cause of organ-specific infection. Transcriptome analysis revealed a high expression of stress-response and amino acid metabolism genes in the plant stems upon *C. ilicicola* infection. Thus, our results suggest that stems prevent *C. ilicicola* infection by activating an organ-specific defense response. It remains to be elucidated how stems sense and exploit specific defense mechanisms to prevent *C. ilicicola* invasion, on which further research is important not only to better understand pathogenetic mechanisms but also for the development of novel genes for disease resistance.

## Data Availability Statement

The original contributions presented in the study are publicly available. This data can be found here: National Center for Biotechnology Information (NCBI) BioProject database under accession number PRJNA781415.

## Author Contributions

C-JJ designed the study. KW and MK performed the experiments and data analyses. MK and C-JJ wrote the manuscript. All authors have read, reviewed, and agreed to the submission of the manuscript.

## Conflict of Interest

The authors declare that the research was conducted in the absence of any commercial or financial relationships that could be construed as a potential conflict of interest.

## Publisher’s Note

All claims expressed in this article are solely those of the authors and do not necessarily represent those of their affiliated organizations, or those of the publisher, the editors and the reviewers. Any product that may be evaluated in this article, or claim that may be made by its manufacturer, is not guaranteed or endorsed by the publisher.

## Footnotes

1
http://systemsbiology.cau.edu.cn/agriGOv2/

2
https://david.ncifcrf.gov/home.jsp

## References

[B1] Abdelsamad N. A. MacIntosh G. C. Leandro L. F. S. (2019). Induction of ethylene inhibits development of soybean sudden death syndrome by inducing defense-related genes and reducing *Fusarium virguliforme* growth. *PLoS One* 2019:14. 10.1371/journal.pone.0215653 31116746 PMC6530837

[B2] Agrios G. N. (1997). *Plant Pathology*, 4th Edn. San Diego, CA: Academic Press.

[B3] Ahmadi B. Masoomi-Aladizgeh F. Shariatpanahi M. E. Azadi P. Keshavarz-Alizadeh M. (2016). Molecular characterization and expression analysis of *SERK1* and *SERK2* in *Brassica napus* L.: implication for microspore embryogenesis and plant regeneration. *Plant Cell Rep.* 35 185–193. 10.1007/s00299-015-1878-6 26449417

[B4] Akamatsu H. Fujii N. Saito T. Sayama A. Matsuda H. Kato M. (2020). Factors affecting red crown rot caused by *Calonectria ilicicola* in soybean cultivation. *J. Gen. Plant Pathol.* 86 363–375.

[B5] Badri D. V. Loyola-Vargas V. M. Du J. Stermitz F. R. Broeckling C. D. Iglesias-Andreu L. (2008). Transcriptome analysis of *Arabidopsis* roots treated with signaling compounds: a focus on signal transduction, metabolic regulation and secretion. *New Phytol.* 179 209–223. 10.1111/j.1469-8137.2008.02458.x 18422893

[B6] Balmer D. Mauch-Mani B. (2013). More beneath the surface? Root *versus* shoot antifungal plant defenses. *Front. Plant Sci.* 4:256. 10.3389/fpls.2013.00256 23874350 PMC3709096

[B7] Bartels S. Lori M. Mbengue M. van Verk M. Klauser D. Hander T. (2013). The family of Peps and their precursors in *Arabidopsis*: differential expression and localization but similar induction of pattern-triggered immune responses. *J. Exp. Bot.* 64 5309–5321. 10.1093/jxb/ert330 24151300

[B8] Bednarek P. Schneider B. Svatoš A. Oldham N. J. Hahlbrock K. (2005). Structural complexity, differential response to infection, and tissue specificity of indolic and phenylpropanoid secondary metabolism in Arabidopsis roots. *Plant Physiol.* 138 1058–1070. 10.1104/pp.104.057794 15923335 PMC1150420

[B9] Bell D. K. Locke B. J. Thompson S. S. (1973). The status of Cylindrocladium black rot of peanut in Georgia since its discovery in 1965. *Plant Dis. Rep.* 57 90–94.

[B10] Bell D. K. Sobers E. K. (1966). A peg, pod, and root necrosis of peanuts caused by a species of *Calonectria*. *Phytopathology* 56 1361–1364.

[B11] Berhin A. de Bellis D. Franke R. B. Buono R. A. Nowack M. K. Nawrath C. (2019). The root cap cuticle: a cell wall structure for seedling establishment and lateral root formation. *Cell* 176 1367–1378. 10.1016/j.cell.2019.01.005 30773319

[B12] Berner D. K. Berggren G. T. Snow J. P. White E. P. (1988). Distribution and management of red crown rot of soybean in Louisiana. *Appl. Agric. Res.* 3 160–166.

[B13] Bolton M. D. Thomma B. P. H. J. Nelson B. D. (2006). *Sclerotinia sclerotiorum* (Lib.) de Bary: biology and molecular traits of a cosmopolitan pathogen. *Mol. Plant Pathol.* 7 1–16. 10.1111/j.1364-3703.2005.00316.x 20507424

[B14] Chu S. Wang J. Zhu Y. Liu S. Zhou X. Zhang H. (2017). An R2R3-type MYB transcription factor, GmMYB29, regulates isoflavone biosynthesis in soybean. *PLoS Genet.* 13:e1006770. 10.1371/journal.pgen.1006770 28489859 PMC5443545

[B15] Day R. B. Shibuya N. Minami E. (2003). Identification and characterization of two new members of the *GRAS* gene family in rice responsive to *N*-acetylchitooligosaccharide elicitor. *Biochim. Biophys. Acta* 1625 261–268. 10.1016/S0167-4781(02)00626-7 12591613

[B16] Dong H. Tan J. Li M. Yu Y. Jia S. Zhang C. (2019). Transcriptome analysis of soybean WRKY TFs in response to *Peronospora manshurica* infection. *Genomics* 111 1412–1422. 10.1016/j.ygeno.2018.09.014 30267765

[B17] Dufresne M. Osbourn A. E. (2001). Definition of tissue-specific and general requirements for plant infection in a phytopathogenic fungus. *Mol. Plant Microbe Interact.* 14 300–307. 11277427 10.1094/MPMI.2001.14.3.300

[B18] Eulgem T. Rushton P. J. Robatzek S. Somssich I. E. (2000). The WRKY superfamily of plant transcription factors. *Trends Plant Sci.* 5 199–206. 10785665 10.1016/s1360-1385(00)01600-9

[B19] Gao L. Bradeen J. M. (2016). Contrasting potato foliage and tuber defense mechanisms against the late blight pathogen *Phytophthora infestans*. *PLoS One* 11:e0159969. 10.1371/journal.pone.0159969 27441721 PMC4956046

[B20] Hermanns M. Slusarenko A. J. Schlaich N. L. (2003). Organ-specificity in a plant disease is determined independently of *R* gene signaling. *Mol. Plant Microbe Interact.* 16 752–759. 12971598 10.1094/MPMI.2003.16.9.752

[B21] Huang D. W. Sherman B. T. Lempicki R. A. (2009). Systematic and integrative analysis of large gene lists using DAVID Bioinformatics Resources. *Nat. Protoc.* 4 44–57. 19131956 10.1038/nprot.2008.211

[B22] Jiang C. J. Sugano S. Ochi S. Kaga A. Ishimoto M. (2020). Evaluation of *Glycine max* and *Glycine soja* for resistance to *Calonectria ilicicola*. *Agronomy* 10:887. 10.3390/agronomy10060887

[B23] Kadotani N. Akagi A. Takatsuji H. Miwa T. Igarashi D. (2016). Exogenous proteinogenic amino acids induce systemic resistance in rice. *BMC Plant Biol.* 16 1–10. 26940322 10.1186/s12870-016-0748-x PMC4778346

[B24] Kim D. Langmead B. Salzberg S. L. (2015). HISAT: a fast spliced aligner with low memory requirements. *Nat. Methods* 12 357–360. 10.1038/nmeth.3317 25751142 PMC4655817

[B25] Kim K. D. Russin J. S. Snow J. P. (1998). Susceptibility to *Calonectria ilicicola* in soybean grown in greenhouse and field. *Korean J. Crop Sci.* 43 239–244.

[B26] Kim K. D. Russin J. S. Snow J. P. Damann K. E. (2001). Toxic culture filtrates produced by *Calonectria ilicicola*, causal agent of red crown rot of soybean. *Phytoparasitica* 29 115–123.

[B27] Krigsvold D. T. Garren K. H. Griffin G. J. (1977). Importance of peanut field cultivation and soybean cropping in the spread of *Cylindrocladium crotalariae* within and among peanut fields. *Plant Dis. Rep.* 61 495–499.

[B28] Lacaze A. Joly D. L. (2020). Structural specificity in plant–filamentous pathogen interactions. *Mol. Plant Pathol.* 21 1513–1525. 10.1111/mpp.12983 32889752 PMC7548998

[B29] le Fevre R. O’Boyle B. Moscou M. J. Schornack S. (2016). Colonization of barley by the broad-host hemibiotrophic pathogen *Phytophthora palmivora* uncovers a leaf development-dependent involvement of *Mlo*. *Mol. Plant Microbe Interact.* 29 385–395. 10.1094/MPMI-12-15-0276-R 26927001

[B30] Liao Y. Smyth G. K. Shi W. (2014). featureCounts: an efficient general purpose program for assigning sequence reads to genomic features. *Bioinformatics* 30 923–930. 10.1093/bioinformatics/btt656 24227677

[B31] Liu X. Yuan L. Xu L. Xu Z. Huang Y. He X. (2013). Over-expression of *GmMYB39* leads to an inhibition of the isoflavonoid biosynthesis in soybean (*Glycine m*ax. L). *Plant Biotechnol. Rep.* 7 445–455. 10.1007/s11816-013-0283-2

[B32] Liu Y. Li Y. Bi Y. Jiang Q. Mao R. Liu Z. (2021). Induction of defense response against Alternaria rot in Zaosu pear fruit by exogenous L-lysine through regulating ROS metabolism and activating defense-related proteins. *Postharvest Biol.Technol.* 179:111567.

[B33] Lynne B. (2016). “Pathogens of Autotrophs,” in *The Fungi*, eds Watkinson S. C. Boddy L. Money N. P. (Cambridge, MA: Academic Press), 245–292. 10.1016/B978-0-12-382034-1.00008-6

[B34] Lyons R. Stiller J. Powell J. Rusu A. Manners J. M. Kazan K. (2015). Fusarium *oxysporum* triggers tissue-specific transcriptional reprogramming in *Arabidopsis thaliana*. *PLoS One* 10:e0121902. 10.1371/journal.pone.0121902 25849296 PMC4388846

[B35] Ma H. S. Liang D. Shuai P. Xia X. L. Yin W. L. (2010). The salt-and drought-inducible poplar GRAS protein SCL7 confers salt and drought tolerance in *Arabidopsis thaliana*. *J. Exp. Bot.* 61 4011–4019. 10.1093/jxb/erq217 20616154 PMC2935874

[B36] Marcel S. Sawers R. Oakeley E. Angliker H. Paszkowski U. (2010). Tissue-adapted invasion strategies of the rice blast fungus *Magnaporthe oryzae*. *Plant Cell* 22 3177–3187. 10.1105/tpc.110.078048 20858844 PMC2965542

[B37] Mayton H. Griffiths H. Simko I. Cheng S. Lorenzen J. de Jong W. (2010). Foliar and tuber late blight resistance in a *Solanum tuberosum* breeding population. *Plant Breed.* 129 197–201. 10.1111/j.1439-0523.2009.01671.x

[B38] Nishi K. (1989). Present situation on Calonectria root rot of soybean (in Japanese). *J. Agric. Sci.* 44 70–75.

[B39] Ochi S. Kuroda T. (2021). Developing a qPCR assay for the quantification of *Calonectria ilicicola* in soil of soybean field. *Trop. Plant Pathol.* 46 186–194. 10.1007/s40858-020-00399-w

[B40] Ochi S. Nakagawa A. (2010). A simple method for long-term cryopreservation of *Calonectria ilicicola* on barley grains. *J. Gen. Plant Pathol.* 76 112–115.

[B41] Ochi S. Yoshida M. Nakagawa A. Natsume M. (2011). Identification and activity of a phytotoxin produced by *Calonectria ilicicola*, the causal agent of soybean red crown rot. *Can. J. Plant Pathol.* 33 347–354. 10.1080/07060661.2011.593558

[B42] Osbourn A. E. (1996). Preformed antimicrobial compounds and plant defense against fungal attack. *Plant Cell* 8 1821–1831. 12239364 10.1105/tpc.8.10.1821 PMC161317

[B43] Padgett G. B. Kuruppu P. U. Russin J. S. (2015). “Red crown rot,” in *Compendium of Soybean Diseases and Pest*, eds Artman G. L. Rupe J. C. Sikora E. J. Domier L. L. Davis J. A. Steffey K. L. (St. Paul, MN: The American Phytopathological Society Press), 79–80.

[B44] Park J. Y. Jin J. Lee Y. W. Kang S. Lee Y. H. (2009). Rice blast fungus (*Magnaporthe oryzae*) infects Arabidopsis via a mechanism distinct from that required for the infection of rice. *Plant Physiol.* 149 474–486. 10.1104/pp.108.129536 18987215 PMC2613700

[B45] Park T. H. Vleeshouwers V. G. A. A. Kim J. B. Hutten R. C. B. Visser R. G. F. (2005). Dissection of foliage and tuber late blight resistance in mapping populations of potato. *Euphytica* 143 75–83. 10.1007/s10681-005-2658-0

[B46] Pataky J. K. Beute M. K. Wynne J. C. Carlson G. A. (1983). Peanut yield, market quality and value reductions due to Cylindrocladium black rot. *Peanut Sci.* 10 62–66. 10.3146/i0095-3679-10-2-5

[B47] Pluemjit N. Sripo-ngam S. Khwanbua E. Chatnaparat T. (2020). Role of Amino Acid Cysteine in the Suppression of Xanthomonas citri pv. glycines Bacterial Pustule Disease of Soybean. *CMU J. Nat. Sci.* 19:833.

[B48] Qiu X. M. Sun Y. Y. Ye X. Y. Li Z. G. (2020). Signaling role of glutamate in plants. *Front. Plant Sci.* 10:1743. 32063909 10.3389/fpls.2019.01743 PMC6999156

[B49] Robinson M. D. McCarthy D. J. Smyth G. K. (2009). edgeR: a Bioconductor package for differential expression analysis of digital gene expression data. *Bioinformatics* 26 139–140. 10.1093/bioinformatics/btp616 19910308 PMC2796818

[B50] Roy K. W. McLean K. S. Lawrence G. W. Patel M. V. Moore W. F. (1989). First report of red crown rot on soybeans in Mississippi. *Plant Dis.* 73:273. 10.1094/PD-73-0273F

[B51] Schäfer W. Yoder O. C. (1994). Organ specificity of fungal pathogens on host and non-host plants. *Physiol. Mol. Plant Pathol.* 45 211–218.

[B52] Schilling L. Matei A. Redkar A. Walbot V. Doehlemann G. (2014). Virulence of the maize smut *Ustilago maydis* is shaped by organ-specific effectors. *Mol. Plant Pathol.* 15 780–789. 10.1111/mpp.12133 25346968 PMC6638905

[B53] Schmutz J. Cannon S. B. Schlueter J. Ma J. Mitros T. Nelson W. (2010). Genome sequence of the palaeopolyploid soybean. *Nature* 463 178–183. 10.1038/nature08670 20075913

[B54] Schreiber C. Slusarenko A. J. Schaffrath U. (2011). Organ identity and environmental conditions determine the effectiveness of nonhost resistance in the interaction between *Arabidopsis thaliana* and *Magnaporthe oryzae*. *Mol. Plant Pathol.* 12 397–402. 10.1111/j.1364-3703.2010.00682.x 21453434 PMC6640388

[B55] Seo S. Nakaho K. Hong S. W. Takahashi H. Shigemori H. Mitsuhara I. (2016). L-Histidine induces resistance in plants to the bacterial pathogen Ralstonia solanacearum partially through the activation of ethylene signaling. *Plant Cell Physiol.* 57 1932–1942. 27335353 10.1093/pcp/pcw114

[B56] Skibbe D. S. Doehlemann G. Fernandes J. Walbot V. (2010). Maize tumors caused by *Ustilago maydis* require organ-specific genes in host and pathogen. *Science* 328 89–92. 10.1126/science.1185775 20360107

[B57] Sugano S. Sugimoto T. Takatsuji H. Jiang C. J. (2013). Induction of resistance to *Phytophthora sojae* in soyabean (*Glycine max*) by salicylic acid and ethylene. *Plant Pathol.* 62 1048–1056. 10.1111/ppa.12011

[B58] Sun Y. Wang M. Mur L. A. J. Shen Q. Guo S. (2020). Unravelling the roles of nitrogen nutrition in plant disease defenses. *Int. J. Mol. Sci.* 21:572. 10.3390/ijms21020572 PMC7014335 31963138

[B59] Tian T. Liu Y. Yan H. You Q. Yi X. Du Z. (2017). agriGO v2.0: a GO analysis toolkit for the agricultural community, 2017 update. *Nucl. Acids Res.* 45 W122–W129. 10.1093/nar/gkx382 28472432 PMC5793732

[B60] Win K. T. Jiang C. J. (2021). A fresh weight-based method for evaluating soybean resistance to red crown rot. *Breed. Sci.* 71 384–389. 34776745 10.1270/jsbbs.20145 PMC8573548

[B61] Yamamoto R. Nakagawa A. Shimada S. Komatsu S. Kanematsu S. (2017). Histopathology of red crown rot of soybean. *J Gen. Plant Pathol.* 83 23–32. 10.1007/s10327-016-0694-3

[B62] Yan J. Wang B. Zhong Y. Yao L. Cheng L. Wu T. (2015). The soybean R2R3 MYB transcription factor *GmMYB100* negatively regulates plant flavonoid biosynthesis. *Plant Mol. Biol.* 89 35–48. 10.1007/s11103-015-0349-3 26231207

[B63] Yang J. Jing X. Wang T. Di J. Chen L. Wang Y. (2020). Overactivation of glutamate consuming pathways in l-glutamate treated tomato fruits lead to resistance against Alternaria Alternata. *Postharvest Biol. Technol.* 169:111311.

[B64] Yariyama S. Ando S. Seo S. Nakaho K. Miyashita S. Kanayama Y. (2019). Exogenous application of l-histidine suppresses bacterial diseases and enhances ethylene production in rice seedlings. *Plant Pathol.* 68 1072–1078.

[B65] Yi J. Derynck M. R. Li X. Telmer P. Marsolais F. Dhaubhadel S. (2010). A single-repeat MYB transcription factor, GmMYB176, regulates *CHS8* gene expression and affects isoflavonoid biosynthesis in soybean. *Plant J.* 62 1019–1034. 10.1111/j.1365-313X.2010.04214.x 20345602

[B66] Zeier J. (2013). New insights into the regulation of plant immunity by amino acid metabolic pathways. *Plant Cell Environ.* 36 2085–2103. 23611692 10.1111/pce.12122

[B67] Zhang Y. Wang H. L. Li Z. Guo H. (2020). Genetic network between leaf senescence and plant immunity: crucial regulatory nodes and new insights. *Plants* 9:495. 10.3390/plants9040495 PMC7238237 32294898

[B68] Zhou Q. Y. Tian A. G. Zou H. F. Xie Z. M. Lei G. Huang J. (2008). Soybean WRKY-type transcription factor genes, *GmWRKY13*, *GmWRKY21*, and *GmWRKY54*, confer differential tolerance to abiotic stresses in transgenic *Arabidopsis* plants. *Plant Biotechnol. J.* 6 486–503. 10.1111/j.1467-7652.2008.00336.x 18384508

