# Supplementary figures and images for: Soybean Hypocotyls Prevent Calonectria ilicicola Invasion by Multi-Layered Defenses
Source: Front Plant Sci. 2022 Jan 24;12:813578. doi: 10.3389/fpls.2021.813578 (PMC8819093; doi:10.3389/fpls.2021.813578)

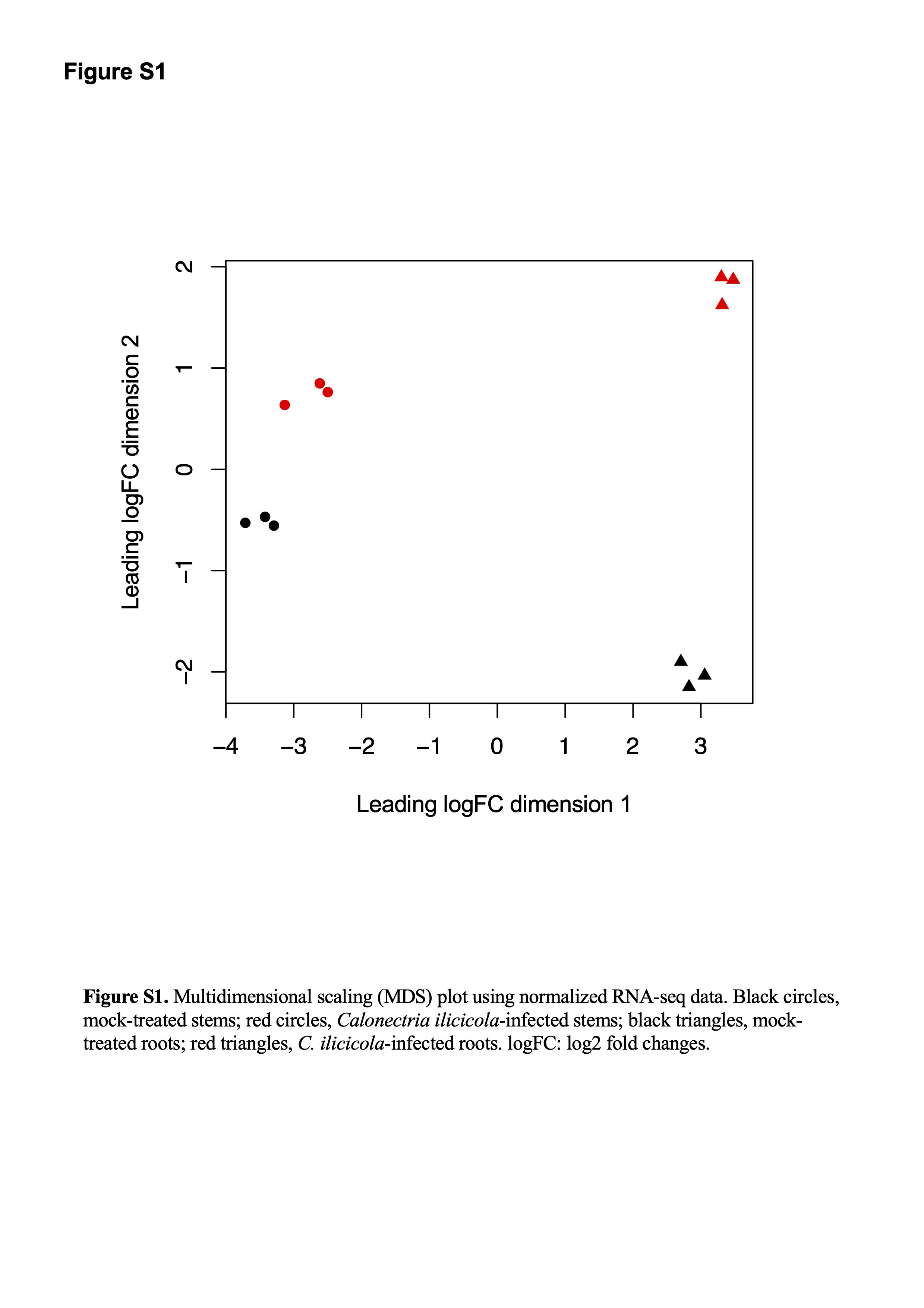

Supplement: Supplementary file 1 [file Image_1.JPEG]

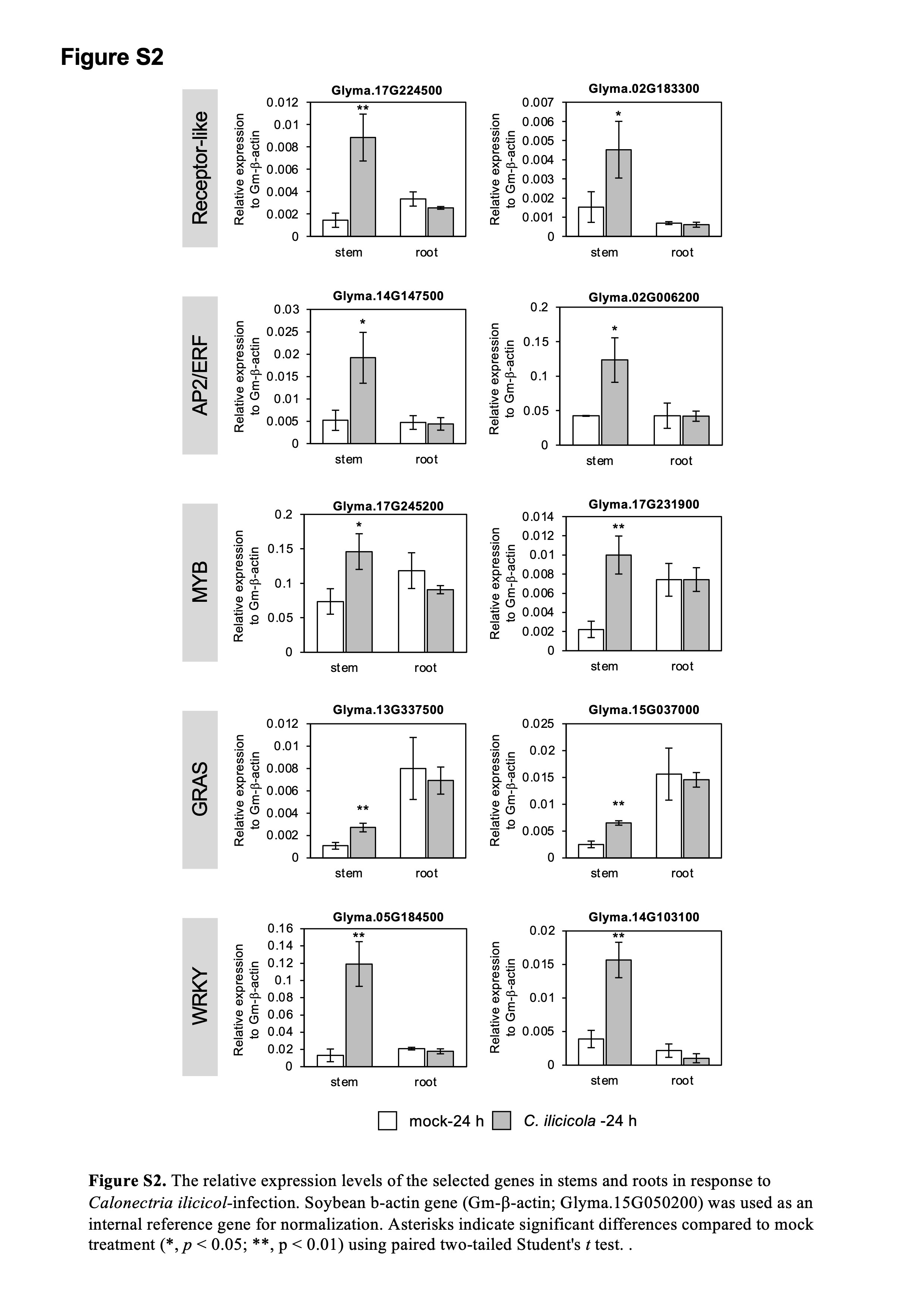

Supplement: Supplementary file 2 [file Image_2.JPEG]

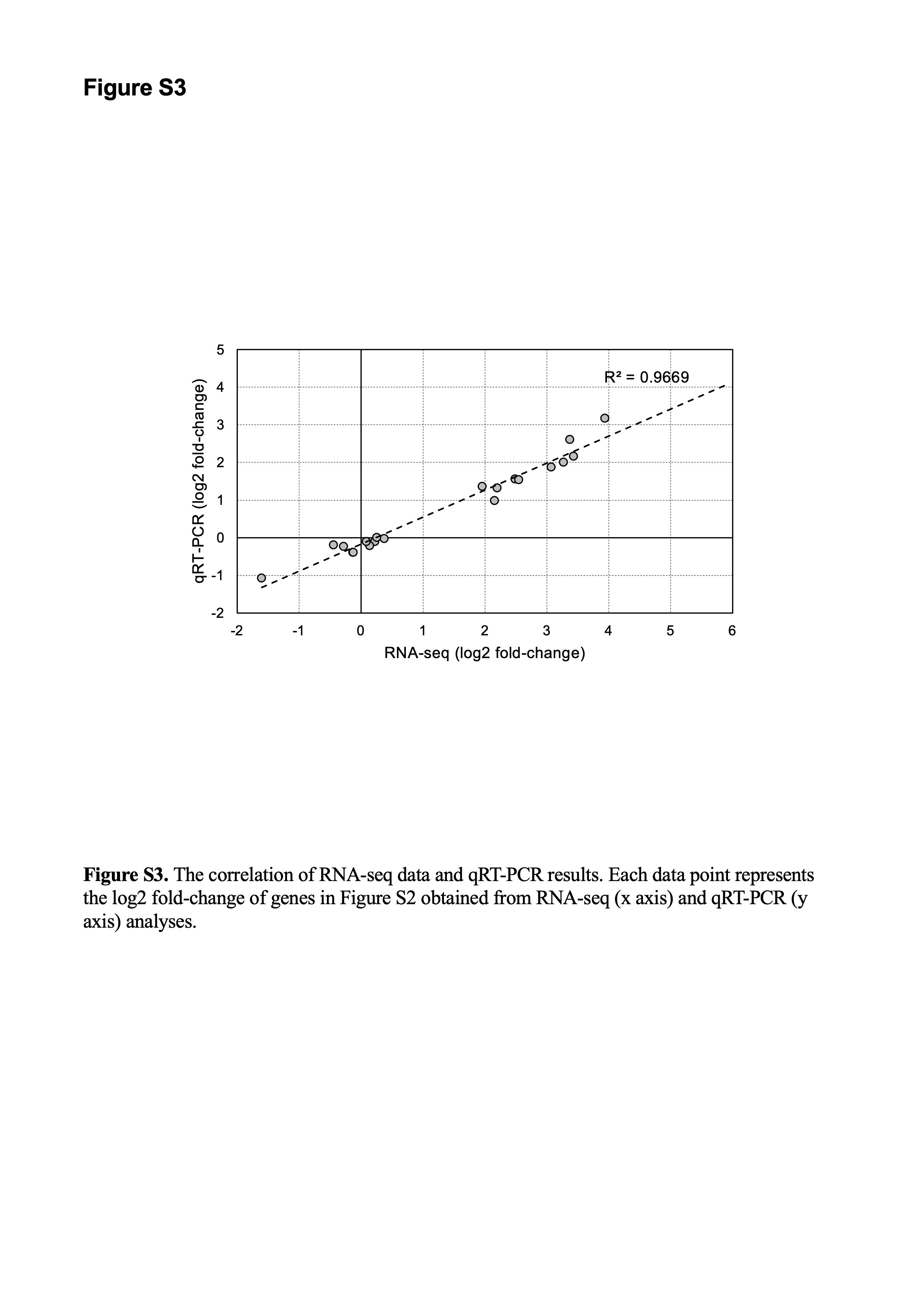

Supplement: Supplementary file 3 [file Image_3.JPEG]
